# Tetany

**Published:** 1908-06-13

**Authors:** Edmund Cautley

**Affiliations:** Physician to the Metropolitan Hospital and the Belgrave Hospital for Children.


### Hospital Clinics.

### TETANY.

By EDMUND CAUTLEY, M.D., F.R.C.P., Physician to the Metropolitan Hospital and the Belgrave Hospital for Children.

I wish to direct your attention to a group of symptoms sufficiently definite to be worthy of description as the definite clinical entity which is named tetany. Although much has been written on the subject, remarkably little is known about its pathology. Tetany was first described by Steinheim, of Altona, in 1830, under the name of "two rare forms of severe rheumatism." Trousseau called it "the rheumatic contraction of nurses," having met with 40 cases in nursing mothers. Dana described it fully in 1831, but the name "tetanie" was first suggested by Corvisart in 1852. More recently the affection has been included in a group of spasmodic diseases under the name of "spasmophilia." Nevertheless we may remain content with the name "tetany," for it gives us a certain amount of information about the affection and commits us to no theory of its causation.

There has been some discussion as to whether simple carpopedal spasm is a variety of tetany. On the whole it is advisable to include the following types, namely:—

1. Clenched fist, with the thumb inturned and flexed, and the fingers flexed over it. This is the variety called carpopedal spasm or "arthrogryposis." The feet are affected, the toes being strongly flexed, with the big toe sometimes abducted and hyperextended, and the heel drawn up. This attitude of the hand is seen in other conditions, notably eclamptic attacks and various affections of the central nervous system.

2. Thumbs strongly flexed and inturned, fingers flexed at the metacarpo-phalangeal joints and hyperextended at the other joints.

3. The "gynæcological hand," to be subsequently described, which is the typical attitude of the hand in tetany.

Tetany may be described as a variety of spasm of the muscles which especially affects the extremities, notably the hands and feet. The spasm is tonic in character; continuous, intermittent, or paroxysmal. It is almost invariably symmetrical, though occasionally limited to one limb. It is of uncertain duration, persists during sleep, and is not associated with loss of consciousness or impairment of intelligence; nor is it due to organic nerve lesions. Frequently the symptoms are latent, slight, and overlooked. In children the most common variety is the intermittent one, associated with rickets, laryngospasm, and generally latent signs. The persistent type is infrequent. The latter is the type most often seen in adults, and possibly, though not probably, differs in its pathology from that of early life.

Certain other affections—namely, the phenomenon of the fist, myotonia, and pseudo-tetanus, possibly also catalepsy, or flexibilitas cerea—are more or less allied to tetany.

The geographical distribution of the affection varies considerably, perhaps on account of climatic conditions, or dependent on the amount of attention attracted to childish ailments. Griffith, in 1850, found only 50 cases, 38 in children, reported in America. Family predisposition may be disregarded, though in some families the children seem more liable than in others. I have known it occur in two brothers. Cases are rather more common in the spring than at other periods of the year, but they occur all the year round. Possibly cold and the bad hygienic conditions of home life in the winter months are predisposing factors.

I have looked up the notes, which I have thought worth keeping, of a few marked cases, and find that of 14, seven were in the first, five in the second, and two in the third year of life. According to Barthez and Sanné, 50 per cent. occur under two years of age, while Griffith gives the percentage as 66. Certainly it is most common in the first two years of life, especially between the ages of six and eighteen months, when rickets is prevalent and the evil effects of erroneous feeding are most frequently seen. Males are much more susceptible than females during the early years of life, though at a later age females are the more liable. Of the 14 cases, 10 were males.

Rickets is very often present. Cheadle found it in every case; Fischl in only 60 per cent. In half my cases it was definite. In two, aged three months, there was no sign of it, and in the others it was doubtfully present. The relationship of rickets to tetany and laryngospasm is an important one, but must not be overestimated. These three affections, sometimes conjointly, occur with unusual frequency in infancy, and there seems little doubt that rickets is a predisposing factor in the ætiology of the spasms. On the other hand, it must be remembered that rickets is very prevalent in infancy, that many cases of severe rickets never have a sign of tetany or laryngospasm, and that children with one or other of the latter complaints may present little or no evidence of rickets. It is, therefore, clearly not the only factor.

The state of the alimentary tract is apparently a much more important factor in the ætiology of the disease. Gastric and enteric disorders are the common exciting causes, perhaps reflexly in some instances, as in painful pyloric spasm, more probably through toxæmia. In many adults tetany is associated with dilatation of the stomach, due to either atony or simple pyloric obstruction. Possibly this is the explanation of some infantile cases, for gastrectasis is common in hand-fed infants, in rickets, and may be secondary to febrile diseases and congenital hypertrophic stenosis of the pylorus. It is not the mere dilatation which causes the spasm, but the toxic effects of decomposing stomach contents.

It is the chronic alimentary disorders which are associated with tetany, rather than the acute attacks, in which toxins are rapidly eliminated. A dilated stomach is a chronic disorder. Cases have been reported in children in association with a dilated large intestine. In one such instance the tetany disappeared after irrigation of the colon, but returned on re-accumulation of the fæcal material (Batty Shaw and Mantain, "Clinical Soc. Trans.," Vol. XXIX.). Langmead has reported similar cases in two boys, aged three and six years, under Garrod, and a girl in her third year under Sturges. Among other possible causes may be mentioned intestinal parasites, delayed menstruation at puberty, masturbation, reflex irritation from various sources, and removal of the thyroid and parathyroids.

Pathologically the spasm must be attributed to hyperexcitability of the nerve cells in the bulb and anterior cornua, of the nerves, and of the muscles, due to the action of some toxin. That the nerve cells in the cord are damaged, nutritionally and functionally, is shown by the muscular weakness and atrophy sometimes present. The cerebral neurons and cranial nerves are occasionally affected. Facial irritability is quite common, and squint may develop. The bilateral symmetry and the uniform character of the spasm indicate a spinal affection. An irregular distribution of the symptoms may be due to special susceptibility of certain nerve cells or inequality in the distribution of the poison.

The poison may be present in the food before ingestion, but is more probably derived from the products of imperfect digestion or bacterial activity. Toxic bodies have been isolated from the stomach contents, but so far tetany has not been produced experimentally by the injection of untreated gastric contents into animals. Probably the poison is most often formed in the intestine, for we almost always find evidence of enteric trouble. Yet tetany is more common in the cold months than in the hot ones, when enteric troubles are the more frequent. The poison may pass out of the stomach and then cause the enteric disturbance. It is curious that tetany, associated with a gastrectasis due to congenital pyloric stenosis, may be induced or exaggerated by lavage.

Stoeltzner has advanced the theory that tetany is due to excess of chloride of calcium. Calcium salts are five times as abundant in cow's milk as in human milk, and many cases of tetany are found to be taking a diet of the former. He states that the addition of chloride or acetate of calcium to the food exaggerates the symptoms, but that adding sodium chloride or magnesium carbonate has no effect. The association with rickets he explains as due to an excess of calcium salts in the blood, because of deficient bone formation. Finkelstein ascribes it to phosphorus or lime, and states that whey aggravates the symptoms. On the other hand, it has been ascribed to deficiency of lime; and Silvestre states that the brain in tetany contains less lime than normal. I think you may take it that we have no reliable evidence that anything in the component parts of pure milk is the primary cause. Still tetany is rare in the breast-fed. Only one of my cases was on the breast, and the mother was taking an excess of beer. In all the other cases the diet was of cow's milk or condensed milk, and usually other articles of food, sufficient to cause intestinal derangements. There is little evidence in support of the theory that it is dependent on the parathyroid or thyroid glands. Thyroid extract appears useless in the treatment, though recovery has followed its exhibition. Kussmaul's theory that it is due to dehydration of the body, especially of the muscles and nerves, can hardly be said to hold water. Some of the children are quite well nourished. The most probable explanation, therefore, is that it is due to the action of a toxin, of intestinal origin, on nervous cells which may be specially susceptible by virtue of malnutrition from rickets or other causes.

In infants the spasm may come on quite suddenly, perhaps during an attack of laryngospasm or general convulsions. More often it develops slowly in the course of some alimentary complaint. It may come on during convalescence. Thus, a boy aged 14 months was admitted to hospital for hæmorrhagic œdema, probably angioneurotic in origin. This was followed by colitis. He was a small, ill-nourished child, with no definite signs of rickets. When convalescent and eating well he developed typical tetany, which lasted three days. Older children may complain of premonitory symptoms, such as headache, general malaise and burning, tingling, numbness, or formication. Sometimes the spasms are only present on waking from sleep. They begin at the periphery and spread upwards. The hands are first affected, and assume the position known as "the gynæcological or obstetrical hand," or "the hand of the accoucheur." The fingers are in the attitude of holding a pen, partially flexed at the metacarpo-phalangeal, and fully extended at the other joints, with their tips approximated. The thumb is fully extended and strongly adducted, its tip applied to the palmar surface of the terminal phalanx of the first or second forefinger; or it may be strongly flexed across the palm. The inner and outer borders of the palm are approximated, hollowing the palm. The wrists are immobile, strongly flexed, tense and shiny, perhaps red, and drawn towards the ulnar side. The forearm is semi-pronated and flexed at the elbow, and the arm is adducted. The feet are rigid in the position of equino-varus, with inversion of the sole and approximation of its inner and outer borders. The sole is contracted longitudinally, and becomes markedly concave, with a median furrow. The toes are extremely flexed and overlap, or are flexed at the metatarso-phalangeal and extended at the other joints; or the big toe is hyperextended and the other toes flexed. The dorsum of the foot is tense, shiny, and perhaps red. Both hands and feet may be a little swollen with œdema. This swelling may be the first sign, though other indications may be elicited. Commonly the hands are alone affected. Next in frequency come the feet. Other muscles of the limbs may be involved. The legs and thighs are sometimes flexed, and the adductors of the thighs may be so strongly contracted as to cause crossing of the legs. Spasm of the neck and back muscles produces retraction and rigidity of the neck and opisthotonos. Dyspnœa may result from spasm of the thoracic muscles. Squint, trismus, and risus sardonicus are sometimes present. The abdominal muscles, the bladder, diaphragm, and, indeed, every muscle of the body, may be involved. In two instances I have noticed marked tremor of the head and neck, and in one of them the hands and arms were similarly affected. Tenderness in the limbs was noted in one case. Pain, due to the cramp-like spasms or to passive extension of the muscles, induces sudden cries.

Tetany alone does not cause fever. Some other explanation of a rise in temperature and increase in the rate of the pulse and breathing must be sought for, and is usually found in the intestinal condition or in the lungs. Variable appetite, more or less vomiting, and loose undigested stools are almost constant. In only one case did I find the stools normal. The knee-jerks are most variable, sometimes normal, often exaggerated, occasionally absent or impossible to obtain on account of the rigidity. Albuminuria is not infrequent. Sometimes drowsiness, or even semi-coma, is present, and indicates toxæmia.

The most important complications are attacks of laryngospasm with cyanosis, general convulsions, enteritis, bronchitis, and broncho-pneumonia. Certain accessory phenomena must be mentioned. Pressure on the internal bicipital groove produces the gynæcological hand. It is called Trousseau's phenomenon, or the "median nerve reflex." Spasm can be induced by pressure on either the blood-vessels or nerves of the limb. In children pronation of the hand, with some adduction of the thumb and fingers, is usually the only response. Chvostek's sign or "facial irritability" is elicited by a tap or pressure on the facial nerve. This causes contraction of the eyelids and mouth and twitching of the side of the face. It is present also in chorea, epilepsy, and hysteria. Erb's sign is an increased irritability of the nerve and muscles to electrical currents, especially galvanism. It may persist for weeks after the symptoms have disappeared. Finkelstein states that it exists, with or without other symptoms, in 30 per cent. of infants fed on cow's milk or whey, and is not present in those fed on human milk, farinaceous food, broth, and eggs. The phenomenon of the phrenics is a name given by Solovieff to violent rhythmical contractions of the diaphragm which he noted in two adults. He ascribed them to irritation of the phrenics by the movements of the heart, with which they were synchronous.

The duration and severity of tetany is most variable. It may last for days, weeks, or months. It may be mild, painless, and continuous, or severe and paroxysmal. It may be limited to one limb, to the hands, or to the extremities, or may affect the whole body. Paroxysms may last a few minutes, or hours. The intermittent or paroxysmal type is the one most often seen in the rachitic, running an acute or subacute course, with short and painful cramps, increased mechanical and electrical reactions, and a tendency to recur on slight provocation. In one instance the first attack occurred in the third year of life, and persisted on and off for three months; and the child had three more attacks in her fourth, fifth, and seventh years, after which I lost sight of her.

The diagnosis is usually easy, and based on the character of the spasm. Latent tetany is associated with rickets, gastro-intestinal disturbance, laryngospasm, convulsions, and the various accessory phenomena. The characteristic attitude of the hand differs markedly from that of carpopedal spasm. In one case the typical hand was present in the first attack and carpopedal spasm in subsequent attacks, apparently due to teething. Tetanus should not give rise to any difficulty, for trismus and risus sardonicus are important signs, and are rare in tetany. Cerebral mischief is unlikely to cause confusion, except in bad cases in early infancy with general rigidity, adductor spasm of the thighs, and exaggerated reflexes, suggesting cerebral spastic diplegia. The association of tremors with rigidity makes it important to exclude the possibility of encephalitis in such cases.* Carpal spasm I have seen in association with cerebral sclerosis in a girl of six months, of syphilitic parentage. She made croaking noises at odd times, clenched the hands and twitched the arms and legs. At 12 months she presented general rickets, head retraction, carpal spasm, variable rigidity of arms and legs, inability to swallow, and a constant wailing, hoarse cry. Attempts to straighten the neck caused respiratory distress or cessation of breathing. The spasmodic condition persisted, with frequent fine tremors and convulsive movements of the arms, and death resulted from bronchitis. Hochsinger has described, as the "phenomenon of the fist," a contraction of the hand induced by pressure on the nerves of the brachial plexus. It is due to imperfect development of the inhibitory centres, and is said only to occur in infants of six to eight weeks of age. Another form of contraction, limited to the hands and not involving the fingers, has been named "myotonia." It is persistent, lasts several weeks, and develops during gastro-intestinal attacks due to auto-intoxication. It occurs during the early weeks of life, chiefly in the autumn, develops gradually, and is unassociated with hyperexcitability of the nerves or muscles, or facial irritability, laryngospasm, or rickets. It disappears with the cause, and requires no special treatment. There does not, in my opinion, seem any sufficient ground for giving this a separate name. It is apparently a mild type of tetany.

Pseudo-tetanus occupies an intermediate position between tetany and tetanus. Few cases have been recorded—nearly all boys between four and ten years of age—and all recovered. The muscles of the jaw, neck, back, and legs are affected by persistent tonic contractions. The masseters and muscles of the back are chiefly involved, thus resembling tetanus. In some no cause is found; others have been associated with intestinal worms or infectious fevers. Usually abrupt in onset, the symptoms quickly assume a mild type. Acute muscular spasms are mild, few, and can be induced by cold, noise, handling, and emotion. The child is not emotional or nervous, remains cheerful and comfortable, in spite of unnatural attitudes, and recovers in one to six weeks. The knee-jerks are active. Violent attacks of laryngospasm and cyanosis may occur. A condition of opisthotonos and recurrent arrest of

* An interesting case of tuberculous tumour of the middle lobe of the cerebellum, simulating tetany, is reported in the Resident Medical Officers' Department on p. 292.—Ed.

breathing has been named by Escherich "*pseudo-tetanus of the newborn,*" and by Snow "*acute recurring respiratory failure.*"

Tetany is not fatal in itself. The prognosis is that of the associated disease. It is bad in severe marasmus, prolonged diarrhœa, bronchitis, broncho-pneumonia, and œdema of the lungs. Tremor is, in my opinion, a bad sign. Drowsiness or semi-coma indicates severe toxæmia. The prognosis must be guarded, because of the liability to death from laryngospasm or general convulsions. Tetany can be prevented by attention to the general health, warmth, proper clothing, and diet. When present, treat the alimentary troubles by calomel, alkalies, bismuth, etc. Chloral per rectum and hot baths relieve the spasm, and bromides are of some value. The underlying cause must be treated. Attend to the diet, get the alimentary tract into proper condition, order rest in bed for a time, keep the room dark and quiet in bad cases; and treat the rachitic condition when the attack is mild, the digestion good, and the patient convalescent.

